# Impact of a hybrid TGfU-Sport Education unit on student motivation in physical education

**DOI:** 10.1371/journal.pone.0179876

**Published:** 2017-06-28

**Authors:** Alexander Gil-Arias, Stephen Harvey, Adrián Cárceles, Alba Práxedes, Fernando Del Villar

**Affiliations:** 1 Faculty of Sport, Catholic University San Antonio of Murcia (UCAM), Murcia, Spain; 2 Ohio University, Athens, Ohio, United States of America; 3 Faculty of Sport Sciences, University of Extremadura, Cáceres, Spain; 4 Area of Physical Education and Sport, Rey Juan Carlos University, Alcorcón, Madrid, Spain; Åbo Akademi University, FINLAND

## Abstract

The Teaching Games for Understanding (TGfU) and Sport Education (SE) pedagogical models share several objectives and pedagogical processes. Despite this seemingly uncanny relationship, few studies have examined the efficacy of a hybrid TGfU/SE pedagogical model, particularly how a teacher’s utilization of such a model impacts on student motivation. The purpose of the current study was to investigate the effect a hybrid TGfU/SE unit, in comparison to direct instruction, on students’ perceptions of various aspects of their motivation to engage in physical education (autonomous motivation, basic psychological needs, enjoyment and intention to be physically active). A crossover design was utilized, using the technique of counterbalancing. One group experienced a hybrid SE/TGfU unit first, followed by a unit of direct instruction. A second group experienced the units in the opposite order. Participants were 55 students. The intervention was conducted over a total of 16 lessons. The hybrid unit was designed according to the characteristics of SE by using seasons, roles, persistent teams, etc. Learning tasks set by the teacher during individual lessons, however, were designed according to the pedagogical principles of TGfU. Student motivation data was generated using validated questionnaires. Results showed that regardless of the order of intervention, the two groups showed significant improvements in autonomy, competence and enjoyment when they were taught using the hybrid model. Instead, in the variables autonomous motivation, relatedness and intention to be physically active there were no significant improvements in one group. These results demonstrate that it is possible to design varied learning situations in which affiliation, leadership and trust are fostered, while tasks are adapted **to** the characteristics of the students. All this can cause greater autonomous motivation, and consequently, perceived competence in the student, a positive image of the sport to practice, and therefore greater enjoyment and to be physically active.

## Introduction

In physical education, teaching has traditionally been undertaken via a direct instruction pedagogical model. In this model, the teacher is the leader of the teaching-learning process and is ultimately responsible for all decisions on the proposed contents and objectives, lesson management and students’ responsibilities [1]. This model is characterized by the teachers’ utilization of blocks of repetitive practice, in which, students must continuously reproduce movements prescribed by the teacher. The direct instruction pedagogical model has been criticized as it decontextualizes sport teaching, since the technical execution is practiced in isolation to an authentic or real game situation [2]. Moreover, it emphasizes a linear, mechanistic and “one-size-fits-all” pedagogical model that has a predominant focus on student psychomotor outcomes at the expense of social and cognitive outcomes [3].

To offer teachers’ alternatives to direct instruction, Metzler [1] proposed seven additional pedagogical models to be used by teachers in physical education. Included among these alternative models was Teaching Games for Understanding (TGfU) [4] and Sport Education (SE) [5]. In both TGfU and SE models, the student is considered an active learner whose needs are considered when teachers’ design learning tasks [6]. Consequently, the student is placed firmly at the centre of the teaching-learning process [7].

Through a teacher using TGfU, the cognitive domain is prioritized, and students learn the tactical aspects of the game by playing the game in small-sided and/or modified/conditioned versions of it that are developmentally appropriate to the learner [8]. In this sense, the *what* (i.e., decision making) comes before the *how* (i.e., skill execution), and the notion that quality game play cannot emerge until the core techniques are mastered is refuted [9]. However, although the cognitive domain is prioritized through the teachers’ skilful task design, technical skills are simultaneously developed alongside tactics in contextualized situations using the pedagogical principles of modification (representation and exaggeration) and tactical complexity [10]. Scholars have argued that through this interaction between the tactical and technical dimensions of play, student motivation in physical education is increased [11].

The SE model is aimed at producing competent, literate and enthusiastic students [12]. According to Kirk [13], it is a well-established and evidence-based pedagogical model where teachers focus on student-centred learning through a cooperative and constructivist pedagogy, which is facilitated by its six features: 1) organising the unit into seasons, 2) students working in persistent teams, 3) formal competition (in developmentally appropriate small-sided, modified/conditioned games), 4) the assignment of roles other than player (i.e., coach, captain, statistician, etc.), 5) record keeping, and 6) festivity (i.e., in a culminating event). Consequently, the authentic learning environment of SE can assist teachers in enhancing student motivation because students have opportunities to socialize, make decisions and enjoy themselves in competitive situations where levels of effort are strongly valued [14].

One theory that can help explain student motivational processes in physical education contexts is Self-Determination Theory (SDT) [15]. SDT proposes that motivation lies along a continuum, in which three levels of self-determination are distinguished [16, 17]: autonomous motivation (participation for the pleasure of carrying out the activity); controlled motivation (participation to achieve other objectives such as social recognition or external rewards) and amotivation (lacking reasons for participating) [15]. Likewise, it establishes three Basic Psychological Needs (BPN): autonomy (desire to commit to an activity due to one’s own choice), competence (desire to interact efficiently with the medium to feel competent) and relatedness (desire to feel part of the group) [15, 16]. The Hierarchical Model of Motivation (HMM) [18] was constructed in order to associate BPN with SDT motivational constructs [19]. According to the HMM the pedagogical model used by the teacher influences the satisfaction of BPN, and consequently, the level of autonomous motivation. This level of self-determined motivation achieved can help predict, positively or negatively, cognitive, affective, and behavioural outcomes. Consequently, those students who experience positive outcomes in physical education such as enjoyment and intention to be physically active are more highly self-determined and autonomously motivated [20, 21] than students who experience more negative outcomes such as boredom. These latter students are more likely to be less self-determined, demonstrate more controlled motivation or amotivation, and be at greater risk of dropping out of the physical activity and sport [22].

Consequently, in physical education it is very important to investigate how the use of different pedagogical models may affect students’ motivation. So far, research on this subject has been limited to comparing pedagogical models such as TGfU and SE to direct instruction. This research has found that when students are taught by a teacher using TGfU [11, 23] or SE [24], the students demonstrate more self-determined (autonomous) motivation when compared to those taught through direct instruction. For example, Jones, Marshall, and Peters [25] compare the effect of direct instruction and TGfU on intrinsic motivation. The results determined that the TGfU group showed significantly higher scores on the six subscales of intrinsic motivation inventory (IMI; perceptions of interest/enjoyment, sport competence, effort/importance, choice, pressure/tension and usefulness) at the conclusion of the unit. Moreover, Wallhead, Gran, andVidoni [26] suggest that prolonged exposure to SE provokes positive change in the satisfying the students’ psychological need for autonomy, competence and relatedness and, consequently a greater intrinsic motivation.

The Teaching Games for Understanding (TGfU) and Sport Education (SE) pedagogical models share several common objectives, concepts and pedagogical processes. Moreover, learning within these models is underpinned by constructivist theories of learning [6]. However, there are also differences between the two models. For example, while SE focuses on forging an authentic and developmentally appropriate sport experience where students take on roles other than that of player, TGfU focuses on the development on the relational aspects of techniques and tactics through appropriate learning task design. It has been advocated that while each model has its own limitations if applied exclusively and in an isolated way [27] a hybrid TGfU/SE model may result higher quality student outcomes [28].

Until now, a limited number of studies have been conducted on student outcomes associated with teachers’ utilization of a hybrid TGfU/SE model. An initial study by Hastie and Curtner-Smith [27] found that the act of teaching through a hybrid model was a complex endeavour, which required the teacher to possess high levels of pedagogical content knowledge. Later studies additionally examined the impact of a hybrid TGfU/SE model on behavioural outcomes such as decision-making and skill execution. For example, two investigations where units of volleyball and soccer were taught using a hybrid model of SE–Invasion Games Competence Model (ICGM; which shares a similar conceptual structure to TGfU) noted significant improvements in both the level of students’ technical execution in decision-making [29, 30]. While these previous studies have reported the positive effects of a hybrid model on psychomotor and cognitive outcomes, there is, however, limited research that has investigated the impact of a teacher’s utilization of hybrid TGfU/SE model on student motivation.

To extend the above work, the purpose of the current study was to investigate the impact of a hybrid TGfU/SE unit, in comparison to direct instruction, on students’ perceptions of various aspects of their motivation to engage in physical education (BPN, autonomous motivation, enjoyment and intention to be physically active). It was hypothesized that students who received the hybrid unit would show higher BPN scores than direct instruction, and consequently higher levels of autonomous motivation, enjoyment and intention to be physically active. We additionally hypothesized that participants who received direct instruction *after* the hybrid unit would demonstrate lower BPN scores, and consequently, lower levels of autonomous motivation, enjoyment and intention to be physically active, compared to the group that experienced the direct instruction *before* the hybrid unit.

## Method

### Design

Moy et al. [23] recently utilized a crossover design to compare one teacher′s use of constraints-led approach (CLA; which has similar pedagogical features to TGfU) to direct instruction and how this impacted pre-service teacher students’ BPN and intrinsic motivation. While their results showed that the pre-service teacher students’ in both groups reported significantly higher scores on all motivational variables after experiencing the CLA when compared to direct instruction, there were two major limitations of this study. First, it was conducted with pre-service teacher students and not school-aged children. Second, the pre-service teacher students’ participated in only one lesson. These factors mean limited conclusions could be drawn from the study.

The current study investigated the impact of a hybrid TGfU/SE unit in comparison to direct instruction on several motivational variables. A crossover design was utilized, using the technique of counterbalancing [23], which allowed the researchers to control the order of presentation of the experimental conditions to neutralize possible effects of learning [31]. One group experienced the hybrid SE/TGfU unit first, followed by direct instruction unit. The second group experienced the units in the opposite order.

### Participants

The current study was conducted in one secondary school in south-eastern Spain. Participants were 55 students (mean age = 15.45, SD = .41, min = 15 years, max = 16 years). 27 were female and 28 were male. All participants were in their 4^th^ year of secondary education and were members to two Secondary Education school classes that received two weekly 50-minute sessions over the course of one week.

Students in both groups had no prior experience with SE or with any of its pedagogical or managerial features (e.g. carrying out different roles such as leading activities, having formal responsibilities for equipment set up and put away, record keeping, officiating, etc.). On the other hand, all the participants had previous experience in different team sports (e.g. handball, basketball and hockey) where their teacher had employed TGfU. The school in which the study took place had enough equipment and space, so that each group of students could have balls, nets, frisbee and cones for both team practices and multiple small-sided competition games.

The teacher of both classes was male and had 15 years of experience in teaching physical education. Moreover, the teacher had significant experience in using TGfU and designing learning tasks using this model. However, he had no previous experience using SE. Consequently, the teacher completed a course of training about the SE model, which stimulated conversations about teaching using a hybrid TGfU/SE model.

The current study was approved of the Ethics Committee of the Catholic University San Antonio of Murcia (Spain) following the guidelines of the Helsinki Declaration. Informed written consent was obtained from the participants and their parents/guardians. All participants were treated in agreement with the ethical guidelines of the American Psychological Association with respect to participant assent, parent/guardian consent, confidentiality and anonymity.

### Data collection and procedures

#### Autonomous motivation

The Spanish version [17] of the Perceived Locus of Causality [32] was used to provide composite scores for autonomous motivation, which added the items developed by Ferriz, González-Cutre and Sicilia [33] to additionally measure integrated regulation. The questionnaire begins with the sentence “I participate in this class of physical education…” and consisted of 24 items. Based on the established distinction in the SDT between autonomous motivation, controlled motivation and amotivation, autonomous motivation was calculated through the mean score of intrinsic regulation (e.g. “Because I enjoy learning new skills”), integrated regulation (e.g. “Because I believe that physical education is according with my values”) and identified regulation (e.g. “Because I can learn skills that could be used in other areas of my life”) [22]. Each type of regulation was composed of four items and previous research in the physical education context has provided support for the factor structure and internal reliability of this measure [34].

#### Basic psychological needs

To assess perceived need satisfaction of the students, the Spanish adaptation of the Basic Psychological Needs in Exercise Scale [35], specific for the context of physical education [36] was used. The questionnaire begins with the sentence, “In my Physical Education classes…” and includes 12 items distributed into three dimensions. Four items measure autonomy (e.g. “I have the opportunity to choose how to perform the exercises”), four items measure competence (e.g. “I carry out the exercises effectively”) and the other four items measure relatedness (e.g. “I feel very comfortable when I do exercise with other colleagues”). Previous research in the physical education context has demonstrated the internal reliability of the instrument [37].

#### Enjoyment

The Spanish version [38] of the Enjoyment/Boredom in Sport Scale [39] was used. The scale has 6 items distributed into two dimensions: enjoyment and boredom. In the present study, we only considered enjoyment, which was measured by three items (e.g. “I usually enjoy physical education”). Previous research in the physical education context has demonstrated the internal reliability of the instrument [21].

#### Intention to be physically active

The intention to be physically active scale [40] was administered to participants. The questionnaire included five items (e.g. “Usually I practice sport in my free time”). Previous research in the physical education context demonstrated the internal reliability of the instrument [40].

The answers to the questionnaires were assessed on a Likert scale ranging from 1 to 5, where one corresponded with the anchor statement *"strongly disagree"* and five with the anchor statement *"strongly agree"*. Before carrying out the current study, the first author contacted with the physical education teacher to inform him of the purpose of the study. Likewise, potential study participants involved were informed about the process that they were going to follow, placing emphasis on the fact that participation was voluntary. The first author was present when the questionnaires were completed. The first author overviewed how to complete the questionnaire and answered any questions that arose during the process. The different questionnaires were completed in the absence of the physical education teacher. The questionnaires were given to all the participants in the same order and it took each participant between 15–20 minutes to complete the questionnaires.

### Intervention

**Fig 1:**
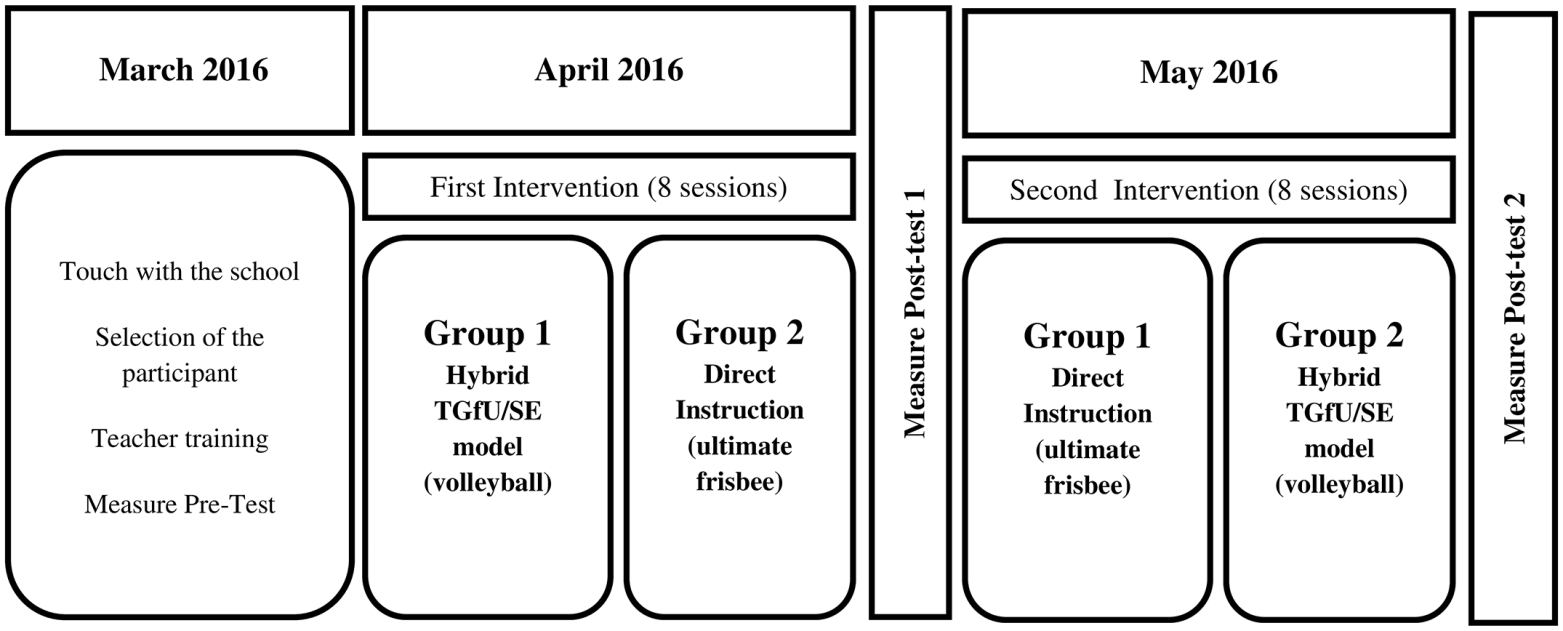
Timeline of the study.

The flow of the intervention can be seen in Fig 1. Before starting the intervention, it was necessary to conduct a period of training with the physical education teacher. The first author led the training process that lasted three weeks. During the first week, the physical education teacher read four papers about SE and four papers about SE and SDT in physical education. In addition, the teacher also read Chapters 1, 5, 7 and 8 of the *Complete guide to sport education* [12], which were related to key features of the SE model, designing seasons to accomplish outcomes, designing competition formats and defining student roles. These documents orientated to the teacher to the philosophical background and six features of SE, as well as the importance of student motivation in physical education context. Once the teacher had read this material, the first author conducted an individual meeting with the teacher to discuss the content of the papers and book chapters. In this meeting, the first author and physical education teacher began discussions about planning a unit of instruction using a hybrid of TGfU/SE model. In week two, the first author and physical education teacher designed the hybrid unit following the structure established by Araújo et al. [29]. In this phase, unit objectives and content were established, as well as the learning tasks for each session (see Table 1). In week three, the physical education teacher carried out two physical education lessons with two different classes of students that did not participate in the actual study. After each teaching session, both of which were observed by the first author, a post-lesson reflection meeting was held to discuss strengths and areas in which both the teacher and first author felt the sessions could be improved. During these reflection meetings, the first author linked discussions to the TGfU/SE model benchmarks seen in Table 2.

**Table 1:** Season plan for the hybrid unit, Sport Education–Teaching Games for Understanding.

Lesson	TGfU component	SE Component
1	Teacher-directed instruction: 1+1 overhand pass (cooperative).	Introduction to the concept of the season–Explain of the model and competition format–Allocation of balanced/mixed ability teams and individual roles–Development of team identity (name, song, colour and picture)—Teacher-directed instruction—within-team practice
2	1vs1 overhand pass. 1vs1+1 overhand pass. 2vs2 overhand pass with questioning	Teacher-directed instruction—within-team practice—Introduction to team roles and responsibilities.
3	2vs2—Serve and overhand pass. 2vs2+1- Serve and overhand pass with questioning.	Teacher-directed instruction—within-team practice—Duty team responsibilities (equipment manager, captain, journalist).
4	3vs3—Serve and overhand pass. 3vs3 –Serve, overhand pass and forearm touch with questioning (e. g. What part of the court is covered by the defence?)	Student-directed instruction: warm-up and cool down—Scrimmages with the opposing teams—First championship for season points—Duty team responsibilities (equipment manager, captain-coach, statistician, and referee).
5	3vs3 –Serve, overhand pass and forearm touch with questioning (e. g. What tendencies do the opponents from the other team have in defence?)	Student-directed instruction: warm-up and cool down–The students have the opportunity to plan some learning task—Scrimmages with the opposing teams—Second championship for season points—Duty team responsibilities (equipment manager, captain-coach, statistician, and referee).
6	3vs3 –Serve, overhand pass and forearms touch with questioning (e.g. Is your position in the field the most appropriate before passing the ball?). 3vs3 –Serve, overhand pass, forearms touch and controlled spike with questioning (e. g. What other attack options were available?)	Student-directed instruction: warm-up and cool down–The students have the opportunity to plan some learning task—Scrimmages with the opposing teams—Third championship for season points—Duty team responsibilities (equipment manager, captain-coach, statistician, and referee).
7	3vs3 –Serve, overhand pass, forearms touch and controlled spike with questioning (e. g. what is your position on the court before making the attack?)	Student-directed instruction: warm-up and cool down–The students have the opportunity to plan some learning task—Scrimmages with the opposing teams—Fourth championship for season points—Duty team responsibilities (equipment manager, captain-coach, statistician, and referee).
8	Culminating event and awards	Culminating event—Festivity.

**Table 2:** Instructional checklist.

Date:	Presence	Absence
1. Group of students go to a designated home area and begin warming up with that group.		
2. Students warm up as a whole class under the direction of the teacher.		
3. Students practice together with their group/team under the direction of a peer leader.		
4. All the tasks are related to the small-sided game that is being taught.		
5. Performance records are kept by students.		
6. Students practice individually or in small groups under the direction of the teacher.		
7. Students perform specialized tasks within their group/team.		
8. Modifications to the full-game were performed.		
9. Student performance scores count toward a formal and public scoring system.		
10. Tasks designed by the teacher were in accordance with the level of student learning.		
11. The teacher observed each team and used the questioning to provoke the reflection.		
12. Student grouping throughout the lesson is variable across tasks.		
13. Student performance scores are not recorded or are recorded in private.		
14. Students employed at least 30 minutes of the session in the practice of modified games.		

Once the teacher training process was completed, pre-test measurements were obtained, after which the intervention began (see Fig 1). The intervention was conducted over a period of eight weeks (two months) for a total of 16 lessons, and focused on the team sports of volleyball and ultimate frisbee. In the first eight lessons, Group 1 experienced the hybrid TGfU/SE model (volleyball), while the Group 2 experienced direct instruction (ultimate frisbee). Once the first intervention was completed, the first post-test data were collected. In the next eight lessons, the Group 1 experienced direct instruction (ultimate frisbee), while Group 2 experienced the hybrid TGfU/SE model (volleyball). Like the end of the first intervention phase, when the second intervention phase was completed, a second round of post-test data were collected. Note that immediately after both direct instruction and TGfU/SE lessons during the intervention, the first author/teacher post-lesson discussions occurred to clarify any issues which may potentially compromise implementation of each of the two pedagogical models. The lesson content for hybrid TGfU/SE model, which is also further described below, is presented in Table 1.

#### Direct instruction

A basic format of sessions focused on direct instruction were as follows: (1) the teacher was the instructional leader of the unit, set the learning goals and tasks, and presented students with a model of desired movement; (2) highly structured sessions, based on the repetition of technical skills; (3) cooperative tasks of two or three people, with the purpose of repeatedly practicing every technical skill–groups were not persistent across lessons; (4) decontextualized game situations where practice is unlikely to generalize to actual game conditions; (5) initial information based on the successful criteria of the execution; (6) prescriptive feedback was provided by the teacher to correct errors [41].

#### Sport Education and Teaching Games for Understanding

The structure of the unit was designed according to the principles and characteristics of Sport Education (seasons, affiliation, formal competition, record keeping, final event and festivity) [42]. This structure can be seen in Table 1. The unit had two phases: (1) Learning phase (lessons 1–3) (2) Formal competition phase (lesson 4–8). In the first lesson of the learning phase, the teacher configured persistent teams using guidelines from Siedentop et al., [12], after which students developed their team identity. For example, they selected a name, image, colour and a chant/song. In the current study, Group 1 consisted of 27 students, who were divided into three groups of 7 students and one group of 6 students. Group 2 consisted of 28 students and 4 groups of 7 students were configured. During the learning phase, students experienced different roles (e.g. coaches, journalist, fitness leader) on a rotating basis, performing different learning tasks within their team to improve their level of competence to carry out these roles. For example, teams participated in a warm up under the guidance of their team’s fitness leader, after which students participated in a team practice, which was instructed by the teacher using direct instruction, but led by the team coach. During the learning phase, equipment was gathered by the equipment manager and the journalists took pictures of their own team participating in the learning tasks. These photos were published on the school website where all members of the educational community could have access.

Once roles were established in the learning phase, team practices were led by the student coaches in the formal competition phase, which began in lesson 4 (see Table 1). During this phase, all teams participated in different competition matches. The formal competition schedule was modified to be developmentally appropriate. For example, equitable participation of all students was guaranteed by ensuring equal playing time for all students. In this formal competition phase the journalist continued to take pictures, however, the nature of some other roles changed. For example, the coach led team practices independent of the teacher, and the statisticians was more prominent as they were responsible for collecting data on the number of games won, earned points, points per player, number of times the ball went to the opposite field after three contacts and rule infringements. Information from the statistician was important because it guided the teaching-learning process towards the SE model objectives, and increased student involvement in the unit. The teacher gathered additional data on team organization, team festivity, team originality, and fair play. His records were made public throughout the unit so that each team could see their progress. After the formal competition phase, a final culminating event was carried out to decide the champions followed by an awards ceremony. Based on the data obtained by the teacher cited above, the following awards were presented: winning team, most original team, most festive team, most organized team, a fair play award and an award for the most valuable player.

The tasks set by the teacher in the learning phase of the SE season were designed according to the characteristics of TGfU. Thus, in each unit learning phase the teacher began the lesson with the presentation of a tactical problem, which students attempted to solve through inquiry and practice. In this regard, the teaching-learning process was developed in a contextualized way, based on the design of modified games that kept the essence of sport as noted in the original aims of SE [43]. In this sense, tasks presented to the students were representative of the reality of the sport, and task modifications were made to adapt the task complexity to the needs of students. In addition, the teacher employed questioning [44] to help guide the students, by way of open-ended questions, towards self-reflection, self-regulation and answers to the tactical problem posed at the start of the lesson. Hence, questioning was utilized to improve the interaction between the technical and tactical decisions required to be effective in the games that were the focus of the intervention [45].

### Instructional and treatment validity

To analyze the influence of a teaching model on different motivational variables, it was necessary to validate that the intervention carried out was consistent with the characteristics of the considered models (hybrid TGfU/SE and direct instruction) in research [46]. A 14-item checklist (see Table 2) was adapted to test the behavioural fidelity of the teacher according to the SE and TGfU models [29, 42]. The first author and one additional observer with experience in instructional models in physical education randomly selected sessions for the assessment of the presence or absence of the items included in Table 2. Items 1, 4, 5, 6, 7, and 9 are characteristics of SE, items 4, 8, 11 and 14 are characteristics of TGfU, while the rest commensurate with direct instruction. A sample of 8 lessons for each model were observed, more than 12.5% the total sample [47]. 100% agreement was reached between the two observers. Each observer therefore confirmed that all key aspects included in the instructional checklist (see Table 2) were performed by the teacher in each of the observed lessons using the two different pedagogical models.

### Data analysis

Data normality was examined through the Shapiro-Wilks test, which led to the use of parametric statistics. Cronbach’s alpha coefficient was employed to calculate the internal reliability for each dependent variable. For each group at each time point, descriptive analyses were calculated. Before analysing the effect of the intervention, it was necessary to complete a MANOVA on the pre-test data to examine if there were statistically significant differences in the dependent variables between the two groups and, therefore, to confirm/disconfirm the homogeneity or heterogeneity of the two groups. This test revealed significant differences between the two experimental groups in some of the dependent variables. Pre-test scores were therefore included as covariates in subsequent analysis [48].

To compare the mean scores of each group in the different dependent variables for each teaching models (e.g. hybrid and traditional), a MANCOVA 2x2 (Test-time x Group) was conducted. In addition, to determine whether any significant differences between the mean scores of the two groups were due to the order that each group experienced the teaching models, a second 2x2 (Test-time x Group) MANCOVA was conducted. In both analyses, pairwise comparisons were analyzed (with Bonferroni correction) when a significant overall effects was found. These subsequent pairwise comparisons enabled the researchers to determine the effect on the interaction between the two measures and between the two groups. Effect sizes were calculated using the partial eta-squared statistic (ηp^2^). The level of statistical significance was established for p ≤ .05, with a confidence interval for differences of 95%. All data analyses were conducted using SPSS v21.0 (Chicago, IL).

## Results

### Preliminary analysis

The results of the initial MANOVA on pre-test scores between groups demonstrated statistically significant differences at multivariate level (Pillai′s Trace = .294; *F*(6, 42) = 2.912; *p* = .018; η_*p*_^2^ = .294; *SP* = .845). In the pairwise comparisons, there were statistically significant differences between groups autonomy (*F* = 12.642; *p* = .001; η_*p*_^2^ = .212); competence (*F* = 6.981; *p* = .011; η_*p*_^2^ = .129), relatedness (*F* = 7.231; *p* = .010; η_*p*_^2^ = .133); in autonomous motivation (*F* = 4.955; *p* = .031; η_*p*_^2^ = .095) and enjoyment (*F* = 10.561; *p* = .002; η_*p*_^2^ = .183). There were no statistically significant differences for the intention to be physically active (*F* = 3.766; *p* = .060; η_*p*_^2^ = .074) (see Table 3).

**Table 3:** Descriptive statistics and inter-group analysis of each dependent variable in pre-test.

	Group 1 Hybrid (first), Traditional (second)		Group 2 Traditional (first), Hybrid (second)		Typical error	*p*	95% CI	ηp2
Dependent Variables	M	SD	M	SD				
Autonomous Motivation	3.91	.71	3.27	1.27	.289	.031	[.062–1.22]	.095
Autonomy	3.48	.75	2.48	1.18	.279	.001	[.431–1.55]	.212
Competence	3.78	.83	3.13	.88	.246	.011	[.155–1.14]	.129
Relatedness	4.33	.77	3.71	.83	.230	0.10	[.156–1.07]	.133
Enjoyment	4.30	.67	3.33	1.37	.300	.002	[.372–1.57]	.183
Intention*	4.20	.85	3.62	1.20	.295	.058	[-.021–1.16]	.074

M = mean; SD = standard deviation.

*Intention means intention to be physically active

### Reliability and descriptive statistics

The descriptive statistics and the internal consistency coefficient (Cronbach’s Alpha) are shown in Table 5. All subscales showed acceptable reliability, exceeding the criterion of .70 [49].

### Intra-group analysis (type of intervention)

The multivariate contrasts showed that there were significant differences between conditions in both Group 1 (Pillai′s Trace = .592; *F*(6, 42) = 10.159; *p* < .001; η_*p*_^2^ = .592; *SP* = 1.00) and Group 2 (Pillai′s Trace = .347; *F*(6, 42) = 3.726; *p* = .005; η_*p*_^2^ = .347; *SP* = .931). More specifically, in Group 1 (hybrid first and traditional second) significant differences in favour of the hybrid condition were found in the following variables: autonomy, competence, relatedness and enjoyment. No significant differences were found for autonomous motivation and intention to be physically active (see Table 4). In the Group 2 (traditional first and hybrid second) significant differences in favour of the hybrid condition were found in the following variables: autonomy, competence, autonomous motivation, enjoyment, and intention to be physically active. No significant differences were found for relatedness (see Table 4). There were significant interaction effects between test-time x group (Pillai′s Trace = .558; *F*(6, 43) = 1.986; *p* < .001; η_*p*_^2^ = .558; *SP* = 1.00).

**Table 4:** Intra-group analysis for both conditions of each dependent variable.

Variables	Group	Intervention 1	Intervention 2	Typical error	*p*	95% CI
Autonomous Motivation	Group 1	Hybrid	Traditional	.173	.600	[-.256 .423]
	Group 2	Traditional	Hybrid	.197	.040	[-.929 -.180]
Autonomy	Group 1	Hybrid	Traditional	.221	<.001	[1.32 2.19]
	Group 2	Traditional	Hybrid	.252	.004	[-1.29 -.380]
Competence	Group 1	Hybrid	Traditional	.154	.002	[.371 1.021]
	Group 2	Traditional	Hybrid	.175	.036	[-.967 -.250]
Relatedness	Group 1	Hybrid	Traditional	.124	.050	[.072 .570]
	Group 2	Traditional	Hybrid	.141	.565	[-.318 .231]
Enjoyment	Group 1	Hybrid	Traditional	.185	<.001	[.810 1.571]
	Group 2	Traditional	Hybrid	.211	<.001	[-1.681 -.841]
Intention*	Group 1	Hybrid	Traditional	.175	.399	[-.204 .504]
	Group 2	Traditional	Hybrid	.200	.033	[-.773 .008]

*Intention means intention to be physically active

### Inter-group analysis (Order)

The multivariate contrasts demonstrated that no significant differences existed in the hybrid condition (Pillai′s Trace = .206; *F*(6, 43) = 1.863; *p* > .05; η_*p*_^2^ = .206; *SP* = .628), while in the direct instruction condition there were significant differences (Pillai′s Trace = .278; *F*(6, 43) = 2.763; *p* = .023; η_*p*_^2^ = .278; *SP* = .824). More specifically, significant differences were found between Group 1 and Group 2, in favour of Group 1 in the hybrid condition, in the following variables: competence, relatedness, enjoyment (see Table 5). Significant differences were found in autonomous motivation between Group 1 and Group 2, in favour of Group 1 in the direct instruction condition (see Table 5). There were no significant interaction effects between test-time x group (Pillai′s Trace = .2017; *F*(6, 43) = 1.986; *p* > .05; η_*p*_^2^ = .217; *SP* = .661).

**Table 5:** Descriptive statistics and inter-group analysis for both conditions of each dependent variable.

			Group 1 Hybrid (first), Traditional (second)		Group 2 Traditional (first), Hybrid (second)		Typical error	*p*	95% CI	ηp2
Dependent Variables	Type of Intervention	α	M	SD	M	SD				
Autonomous Motivation	Hybrid	.92	4.26	.50	3.30	1.34	.236	.041	[.021 .971]	.084
	Traditional	.90	4.17	.47	2.74	1.42	.259	.001	[.360 1.399]	.194
Autonomy	Hybrid	.92	3.96	.76	3.29	.87	.255	.138	[-.128 .896]	.045
	Traditional	.91	2.22	1.04	2.38	.97	.329	.293	[1.012 .312]	.023
Competence	Hybrid	.92	4.23	.58	3.41	.86	.219	.022	[.080 .960]	.105
	Traditional	.86	3.55	.79	2.84	1.12	.255	.166	[-.154 .871]	.040
Relatedness	Hybrid	.84	4.63	.42	3.77	.81	.198	.002	[.238 1.033]	.177
	Traditional	.91	4.29	.59	3.77	.92	.237	.332	[-.244 .708]	.020
Enjoyment	Hybrid	.96	4.62	.46	3.98	.74	.187	.016	[.091 .842]	.115
	Traditional	.91	3.46	.89	2.77	1.27	.299	.560	[-.426 .778]	.007
Intention*	Hybrid	.92	4.32	.80	3.44	1.41	.317	.112	[-.124 1.151]	.052
	Traditional	.90	4.17	.92	3.08	1.48	.369	.072	[-.064 1.421]	.066

M = mean; SD = standard deviation; α = Cronbach's alpha

*Intention means intention to be physically active

## Discussion

The purpose of the current study was to investigate the effect a hybrid TGfU/SE unit, in comparison to direct instruction, on students’ perceptions of various aspects of their motivation to engage in physical education (BPN, autonomous motivation, enjoyment and intention to be physically active). A counterbalanced design was utilized, from which, the order of presentation of the experimental conditions allowed to neutralize possible learning effects. Our first hypothesis was that students who received the hybrid unit would show higher BPN scores than direct instruction, and consequently higher levels of autonomous motivation, enjoyment and intention to be physically active.

The results showed that participants in both groups exhibited significantly greater autonomy when they were taught by the teacher using a hybrid TGfU/SE unit. The increase in students’ perception of autonomy observed in this current study has been reported in previous studies when students have been taught via TGfU [11] and/or SE [26]. In this unit, the students had to assume different responsibilities, and were empowered by the teacher because were provided with the opportunity to solve specific tactical problems. Thus, there was an increase in the perception of autonomy, compared with direct instruction, where the teacher is at the centre of teaching/learning and students reproduce movements prescribed by the teacher.

In addition, both groups recorded significantly higher competence scores after completing a hybrid TGfU/SE unit. This result is consistent with other studies where increases in perceived competence were noted after students received an intervention based on TGfU [11] or SE [50, 51]. Moreover, previous studies have found that a hybrid TGfU/SE unit resulted in improvements in student competence because this hybrid model developed students’ tactical awareness and skill performance [29, 52, 30]. It is our contention that the teachers use of the SE model provides students with the opportunity to carry out those roles that best fit their interests and personal strengths, thus facilitating students’ perceived success [5]. Likewise, the physical education teacher’s utilization of TGfU to design authentic tasks linked to the real game that are appropriately modified in terms of representation, exaggeration and tactically complexity, was also a potential feature of the students’ perceived success and competence in the hybrid unit [53].

Only Group 1 obtained significant differences between hybrid TGfU/SE unit and direct instruction in relatedness. Previous studies have been reported that using either a TGfU [11] and/or SE model [24] increases feelings of unity among members. More specifically, in SE the students are organized into groups that are persistent for the entire season, which results in positive feelings of affiliation and social connection to other group members [54]. TGfU is a pedagogical model in which students must solve tactical problems in collaboration with peers. This necessitates teachers use of questioning to prompt an exchange and debate of ideas among group members, which can potentially increase students’ sense of unity [55]. The affiliation process and building social relationships takes time to develop throughout the season. Therefore, it is possible that the lack of significant changes in this variable for the Group 2 was a consequence of that group feeling the intervention was not sufficient to observe changes in this variable [37, 56].

However, Group 2 showed greater autonomous motivation in the hybrid TGfU/SE unit compared to direct instruction. The results are consistent with previous studies, which found that compared to direct instruction, students taught through TGfU [25] or SE showed higher autonomous motivation [50, 24]. In this case, the teacher adopts pedagogy in TGfU/SE aimed towards satisfying the BPN of students, which consequently increases autonomous motivation [15, 57]. The increase in autonomous motivation, as a result of satisfaction of BPN, has additional positive consequences in physical education, such as greater enjoyment and intention to be physically active, differences which were also found for Group 2 [58]. These results are consistent with other studies, in which the students expressed a high degree of enjoyment in physical education when they experienced a unit based on SE [51] and/or TGfU models [59]. This increase in autonomous motivation is considered important because it gives information about the pleasure and satisfaction of students in physical education, which may be related with a stronger learning and greater academic outcomes [15, 22]. In the hybrid TGfU/SE model, students are at the centre of the teaching-learning process. As a result, it could be argued that students in Group 2 perceived greater social recognition of their actions and, consequently, experienced greater enjoyment and satisfaction then members of Group 1.

On the other hand, it is noteworthy that in Group 1 no significant improvements were obtained in autonomous motivation. These results are consistent with previous studies, which found that the satisfaction of the BPN did not consequentially result in a significant increase in autonomous motivation [37, 60]. This may be because autonomous motivation was already high at the beginning of the study, and a likely ceiling effect occurred for this variable.

We additionally hypothesized that participants who received direct instruction *after* the hybrid unit would demonstrate lower BPN scores, and consequently, lower levels of autonomous motivation, enjoyment and intention to be physically active, compared to the group that experienced the direct instruction *before* the hybrid unit. The results do not confirm support for this hypothesis because participants who received direct instruction *after* hybrid model did not show significantly lower scores in all dependent variables, compared to the group receiving direct instruction *before* the hybrid unit. These findings are different to previous studies. For example, Moy et al., [23] found that the group that experienced the traditional teaching approach after the CLA (constraints-led approach) reported statistically significantly lower motivation subscale mean scores compared to the group that experienced the traditional teaching approach before the CLA. The results of the current study may be due to the particular characteristics of this study, as well as the nature of students Group 1. First, our study was different to Moy et al., [23], in that pre-test measurements were obtained. Research has shown that this is an important factor to consider when utilizing crossover design because students within each existing groups may differ markedly and this may affect the ultimate reporting of results [61]. Second, students in Group 1 had average scores that were relatively higher than Group 2 on all variables and, therefore, may already have a greater motivation for physical education, regardless of the teaching model utilized by the teacher.

We can note several strengths within the current study. First, we utilized a robust counterbalancing technique within our design, taking pre-test scores from both groups, as well as measurements after both the hybrid TGfU/SE and direct instruction units. Second, we controlled for pre-test differences in our analyses, and so we utilized a MANCOVA, which is also preferable when you have been unable to randomly assign your participants to the different groups, but instead have had to use existing groups [48]. Third, we measured and reported teacher fidelity to each of the models. Thus, we can report our results in the knowledge that contamination effects were not present as the same teacher delivered both models.

Although the results may indicate the positive effect of the hybrid model on BPN, autonomous motivation, enjoyment and intention to be physically active, the results should be taken with caution because it is a preliminary study with a small sample. Consequently, future research can extend the current sample by, for example, increasing the number of physical education teachers at different schools, which would provide more power to detect significant differences [42]. Also, we utilized a crossover design where we examined changes in class groups already established. This resulted in different pre-test scores, which may have influenced subsequent analyses, although we did control for this by using a covariate in our analysis. However, it would be interesting for researchers to use analysis techniques commensurate with a blocking design, which enables students to be stratified in groups in the analysis by for example skill level, which reduces issues with heterogeneity within intact groups [62]. This could be also utilized alongside the counterbalancing technique we used in this current study. Note that the TGfU/SE unit presented in this current study was applied according to the reality of physical education in a Spanish context, where units of physical education are limited to 8–10 lessons [63]. In this sense, we conducted an ecologically valid intervention [64]. Despite this, future studies it may be preferable to extend the length and duration of the current TGfU/SE unit to 15–20 lessons, which would be commensurate with the suggested length of an SE season [12], and season lengths documented in previous research studies [42]. In addition, we would like to emphasize that unlike previous studies with a similar research design, where the intervention was performed around the same activity/sport [23], the current intervention was carried out within two different activity areas/sports (one invasion game and one net game). This occurred because the researchers did not want to alter the schedule of units already planned by the physical education teacher. The examination of additional variables beyond motivation is also warranted (e.g., game performance, skill development, physical activity), especially given the holistic nature of the intention of the hybrid TGfU/SE model [42]. Moreover, the extent to which the teacher utilizes autonomous and controlling behaviours could be examined, to provide a potentially richer contextual picture of why certain groups may not demonstrate similar changes in motivation to others [65]. Finally, it would also be interesting to include qualitative data on student perceptions. This will allow us to triangulate our findings, and provide richer information on motivational changes experienced by students when they experience units taught through different models.

## Conclusion

In conclusion, findings from our intra-group comparison show that a hybrid model of TGfU/SE stimulated increases in autonomy, relatedness, competence, autonomous motivation, enjoyment and intention to be physically active compared within direct instruction. These results reinforce the idea that by utilizing a combination of two pedagogical models (i.e., TGfU and SE) that a priori may be different, it is possible to design varied situations learning in affiliation, leadership and trust are fostered, while tasks are adapted to the characteristics of the students. Within this hybrid unit, the design of modified games is key because it allows simplifying the demands of a sport to increase student success. This can causes greater perceived competence in the student, a positive image of the sport to practice, and therefore higher levels enjoyment and greater adherence to practice.

However, our inter-group comparisons did not stimulate the same effects, largely due Group 1 reporting higher scores than Group 2 on the motivational variables examined. Therefore, future studies should utilize an experimental design where pre-test scores with already existing groups can be additionally controlled still further than we were able to in this study. Moreover, future studies should look to extend the current study beyond one teacher in one school context, complete the hybrid TGfU/SE unit over a longer duration, and measure additional variables beyond student motivation to triangulate evidence of student learning.
